# Evolution of Homeologous Gene Expression in Polyploid Wheat

**DOI:** 10.3390/genes11121401

**Published:** 2020-11-25

**Authors:** Na Zhao, Qianli Dong, Brian D. Nadon, Xiaoyang Ding, Xutong Wang, Yuzhu Dong, Bao Liu, Scott A. Jackson, Chunming Xu

**Affiliations:** 1 Department of Agronomy, Jilin Agricultural University, Changchun 130118, China; naz@jlau.edu.cn; 2 Center for Applied Genetic Technologies, University of Georgia, Athens, GA 30602, USA; brian.nadon@uga.edu; 3 Key Laboratory of Molecular Epigenetics of the Ministry of Education (MOE), Northeast Normal University, Changchun 130024, China; dongql043@nenu.edu.cn (Q.D.); wang3283@purdue.edu (X.W.); dyz7802@163.com (Y.D.); baoliu@nenu.edu.cn (B.L.); 4 Soybean Research Institute, Jilin Academy of Agricultural Sciences, Changchun 130033, China; dingxy@cjass.com; 5 Bayer Crop Science, Chesterfield, MO 63017, USA

**Keywords:** polyploidy, transcriptome, differentially expressed gene, wheat, whole genome duplication

## Abstract

Polyploidization has played a prominent role in the evolutionary history of plants. Two recent and sequential allopolyploidization events have resulted in the formation of wheat species with different ploidies, and which provide a model to study the effects of polyploidization on the evolution of gene expression. In this study, we identified differentially expressed genes (DEGs) between four BBAA tetraploid wheats of three different ploidy backgrounds. DEGs were found to be unevenly distributed among functional categories and duplication modes. We observed more DEGs in the extracted tetraploid wheat (ETW) than in natural tetraploid wheats (TD and TTR13) as compared to a synthetic tetraploid (AT2). Furthermore, DEGs showed higher *Ka/Ks* ratios than those that did not show expression changes (non-DEGs) between genotypes, indicating DEGs and non-DEGs experienced different selection pressures. For A-B homeolog pairs with DEGs, most of them had only one differentially expressed copy, however, when both copies of a homeolog pair were DEGs, the A and B copies were more likely to be regulated to the same direction. Our results suggest that both *cis*- and inter-subgenome *trans*-regulatory changes are important drivers in the evolution of homeologous gene expression in polyploid wheat, with ploidy playing a significant role in the process.

## 1. Introduction

Polyploidy, or whole genome duplication (WGD), is a ubiquitous feature in the evolutionary history of angiosperms [1,2]. Allopolyploidy, the joining of two evolutionarily divergent genomes, in particular, has played a crucial role in diversification and speciation among vascular plants [3]. Allopolyploid species show wider geographical distributions, occupy more diverse ecological habitats, and display broader adaptability to various conditions than their diploid progenitors [4].

Extensive gene expression changes following allopolyploidization have been reported in artificially synthesized and natural polyploid species of various taxa, including *Arabidopsis* [5], *Tragopogon* [6], *Gossypium* [7], *Oryza* [8], *Triticum* [9,10,11], and others [12,13]. These changes include patterns such as non-additive expression and expression dominance. Recently, some studies have focused on the allelic and homeologous expression changes in polyploid species. Homeologous expression bias and divergence have been found to increase over time in cotton, rice, and wheat [7,8,10,11]. In tetraploid cotton species, expression level dominance is primarily due to upregulation or downregulation of the homeolog expression of the “non-dominant” parent, which suggests the regulation of gene expression changes in polyploids involves complex *cis*- and *trans*-regulation and interactions [7]. In wheat, newly synthesized and natural tetraploid species showed biased homeolog expression, largely set by parental genome divergences [11]. This was shown to occur rapidly upon allotetraploid formation, and this expression asymmetry was further reinforced during evolution and domestication [11]. Because allopolyploid reference genomes are still quite rare, these studies have been restricted to examining total expression levels (all homeologs summed) or expression ratios between homeologs. However, currently, little is known about the effect of different ploidy levels on the evolution of gene expression of different subgenomes. The recent releases of polyploid reference genomes—such as cotton, peanut, tetraploid wheat, and even hexaploid wheat—have made it possible to do these analyses in a more refined manner [14,15,16,17].

**Figure 1:**
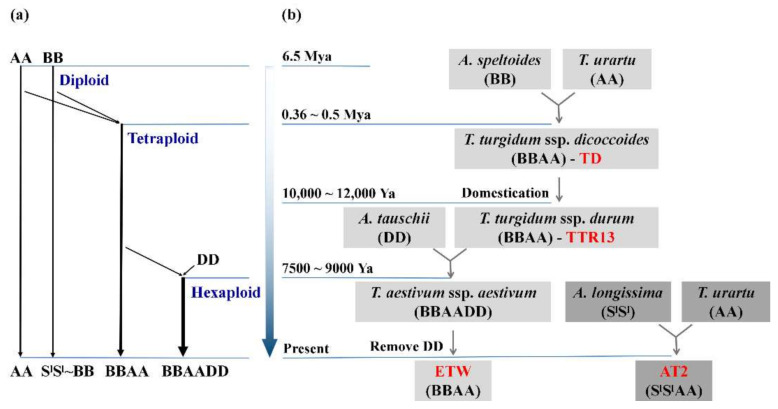
Diagram of wheat ploidy history and targeted genotypes. (**a**) Timeline of the two allopolyploidization events in wheat evolutionary history that resulted in three different ploidy levels, diploid, tetraploid, and hexaploidy (Marcussen et al., 2014) [18]. (**b**) The four tetraploid wheats (red color) used in this study and their relationships in different ploidy backgrounds. In synthetic tetraploid wheat (AT2, S^l^S^l^AA), both AA and BB (S^l^~B) evolved in the diploid background; in wild tetraploid wheat (TD, BBAA) and domesticated tetraploid wheat (TTR13, BBAA), both AA and BB evolved in tetraploid background after the whole genome duplication 0.36–0.5 Mya; In the extracted tetraploid wheat (ETW, BBAA), both AA and BB evolved in a hexaploid background before being extracted.

The *Triticum-Aegilops* complex contains species of different ploidy levels. Thus, it provides an excellent system to study the impact of differing ploidy levels on the evolution of gene expression. There are three different ploidies in these wheat species: diploid, tetraploid, and hexaploid wheat. The two diploid wheat genomes, A and B, diverged from a common progenitor ~6.5 million years ago (Mya) [18]. The wild tetraploid wheat *T. turgidum* L. ssp. *dicoccoides* (BBAA) was formed via allotetraploidization about 0.5 Mya and through domestication produced a new subspecies, *T. turgidum* L. ssp. *durum* (BBAA) [18,19]. Subsequently, a single or multiple allohexaploidization event(s) between *T. turgidum* ssp. *durum* (BBAA) and goat-grass *Aegilops tauschii* (DD) led to the establishment of hexaploid wheat, *T. aestivum* (BBAADD), which occurred less than 10,000 years ago [18,20] (Figure 1a,b). Thus, the AA and BB genomes have evolved independently in parallel in different species with three different ploidy levels for about 8000–10,000 years. In previous studies, the BBAA subgenomes were ‘extracted’ from hexaploid wheat by backcrossing tetraploid wheat TTR13 to hexaploid bread wheat *T. aestivum* L. (cv TAA10) as the recurrent parent [21]. The resulting individuals were then propagated via self-pollination (to eliminate the D-subgenome chromosomes) to form a new BBAA, ‘extracted’ tetraploid wheat (ETW) accessions [21]. Together with a synthetic tetraploid wheat, these tetraploid wheats present an ideal system to study gene expression changes in AA and BB genomes that have evolved in different ploidy levels [11].

All flowering plants have undergone at least two rounds of WGD: one in the common ancestor of seed plants and the second in the common ancestor of angiosperms [1]. Many plant species have experienced additional subsequent WGDs. Fractionation, a process whereby redundant gene copies are deleted and remaining genes are reordered often follows these WGDs [22]. In some lineages, most duplicate genes are lost post-WGD and only a few are retained in duplicate status. The rate of deletion or retention of duplicate genes following WGD is often biased between subgenomes and unevenly distributed among different functional categories or gene families [23,24]. The legacies of ancient WGDs and subsequent evolution can be identified through analysis of within- and between-chromosome collinearity in a diploid genome [25]. The two diploid wheat genomes AA and BB have undergone multiple rounds of early WGDs during their evolution [1,26]. Examination of collinearity of duplicate genes within each diploid genome reveals that genes can be classified into five categories: singleton (no duplicate), dispersed (duplicated in different places along the genome), proximal (duplicated, but close to the original copy), tandem (duplicated and directly adjacent), and WGD/segmental duplicates (several genes duplicated in a similar order) [27]. The evolution of genes in these five categories, conservation and expression, following tetraploidization and hexaploidization in wheat is an outstanding question.

In this study, we compared the gene expression of four BBAA tetraploid wheats in which the AA and BB genomes, either separately or together, came from three different ploidy backgrounds: diploid (AA and S^1^S^1^, from a S^l^S^l^ × AA cross mimicking BB × AA in a synthetic tetraploid AT2), tetraploid (AA and BB in wild tetraploid and domesticated tetraploid wheat), and hexaploid (AA and BB in an extracted tetraploid wheat) to investigate the evolution of gene expression associated with ploidy level changes in wheat. We explored differences in the rate of evolution between DEG (differentially expressed genes) and non-DEG in different types of wheat in this context. Furthermore, we examined associations between differential gene expression and putative functions, duplication modes (singleton, dispersed, proximal, tandem, and WGD), and regulatory mechanisms.

## 2. Materials and Methods 

### 2.1. Plant Materials and RNA-Seq Data

The transcriptome data are from a previously published study but re-analyzed in this study [11]. All sequence data were downloaded from the NCBI SRA database under the accession number PRJNA272886. RNA-seq data of four wheat genotypes were included: the newly synthesized tetraploid wheat (AT2: S^l^S^l^AA, for the evolution under diploid level), wild and domesticated tetraploid wheat (TD and TTR13: BBAA, for evolution in a tetraploid nucleus) and the “extracted” tetraploid wheat from the hexaploid bread wheat (ETW: BBAA) [10,11] (Figure 1a,b). For each genotype, RNA-seq data of two tissues, young leaves and young inflorescences, were used for analysis.

### 2.2. Preprocessing and Mapping of RNA-Seq Data

All raw RNA-seq data were mapped to the genome sequences of a tetraploid wheat (*T. turgidum* ssp. *dicoccoides*, “emmer wheat”) [16] using STAR [28] allowing each read to map to at most 1 location (--outFilterMultimapNmax 1 option). The number of reads mapped to each defined gene region were counted. The aligned files of different replicates and tissues for each genotype were merged into one file for calculating coverage of genes. The per-base mapping depth was calculated in exon regions of primary transcripts for each genotype with BEDTools, BEDOPS and custom Perl scripts [29,30].

### 2.3. Gene Expression Analysis

To mitigate the effects of sequence variation between genotypes on mapping and expression analysis, we kept only genes where 85% of the exon regions (primary transcripts) were covered by uniquely mapped reads (the depth of 85% exon regions were >1 read) in all genotypes. For all genes in either AA or BB genome, gene expression values were normalized among all samples, and then gene expression values for the same tissue compared between different genotypes using DESeq2 [31]. Genes with fold change of expression value >2 and FDR adjusted *p*-value < 0.05 between genotypes were classified as differentially expressed genes (DEGs) in each comparison.

### 2.4. Calculating Ka/Ks Ratio

All raw RNA-seq reads were filtered by “Trim Galore” to remove low quality (<20) and short reads (<75 bp). For each genotype the combined, cleaned data of all replicates and tissues were mapped to the coding DNA sequences (CDS) of primary transcripts of emmer wheat genes by BWA-mem with default parameters [32]. The single nucleotide polymorphisms (SNPs) between each genotype and the reference sequence were called by a SAMtools and BCFtools [33]. Next, alternative sequences for each genotype were created by replacing the reference allele with the alternative allele for each called SNP between the reference and the alternative genotype. For each gene, the newly created coding DNA sequences between two genotypes were then aligned by “Clustalw” and the *Ka* and *Ks* were calculated through the ‘Bio::Align::DNAStatistics’ BioPerl module in a custom Perl script [34]. Genes without any synonymous mutations between genotypes were removed from further analysis. For each pair of genotypes, the *Ka/Ks* ratios for DEGs and non-DEGs were calculated and compared in R.

### 2.5. Classification of Genes in Different Duplicate Status within AA or BB Genome

Homology searches were performed within and between AA and BB genomes for all primary protein sequences using an all-by-all blastp with a cutoff of 1E-10. The blastp result was analyzed by MCScanX [27] with default parameters for collinear block detection. Genes from either genome, AA or BB, were classified into five different categories: singleton, dispersed, proximal, tandem, and WGD/segmental with the “duplicate_gene_classifier” tool in MCScanX.

### 2.6. GO and Pfam Enrichment Analysis

Protein sequences of all genes from the reference genome were analyzed by InterProScan (version 5.26–65.0) to obtain predicted Pfam and GO annotations [35]. The *p*-values for enrichment in GO categories and protein families were calculated using the hypergeometric method and the *p*-values were adjusted by the FDR method (*q*-values) [36].

## 3. Results

### 3.1. Expression Changes in AA and BB Genomes from Different Ploidy Backgrounds

**Figure 2:**
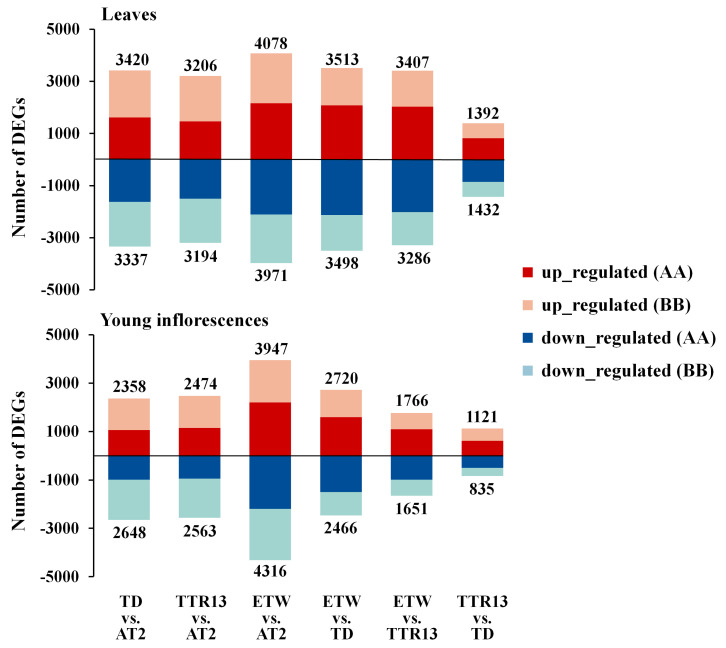
Analysis of differentially expressed genes (DEGs). Number and direction of change of DEGs identified in comparisons between different wheat genotypes in leaves (upper panel) and young inflorescences (lower panel). The total number of upregulated and downregulated genes are shown at top and bottom of x-axis for each comparison. The proportions of AA and BB upregulated DEGs are shown in red and beige, respectively; The proportions of AA and BB downregulated DEGs were shown in dark blue and light blue, respectively.

In this study, for simplicity, the term ‘genome’ and ‘subgenome’ are used interchangeably. Previously published RNA-seq data from two tissues of four different BBAA tetraploid wheat (Figure 1a,b) were obtained [11]. The RNA-seq reads were mapped to the recently released wild tetraploid wheat reference genome (*T. turgidum* ssp. *dicoccoides*) and the expression of genes were obtained (Table S1 and Supporting Dataset 1). In total, 27,097 genes were analyzed, 16,526 and 10,571 genes from A and B genomes, respectively. A dendrogram of gene expression showed that samples clustered into two large groups by tissue, and further into clusters of genotypes (Figure S1). Differentially expressed genes (DEGs) were identified by comparing expression values (FDR adjusted *p*-value < 0.05 and >2-fold change in expression) between genotypes. There were significantly more DEGs identified in ETW (leaves: 8049/29.70%; young inflorescences: 8263/30.49%) than TD (leaves: 6757/24.94%; young inflorescences: 5006/18.47%) and TTR13 (leaves: 6400/23.62%; young inflorescences: 5037/18.59%) when each was compared to AT2 (Figure 2, Tables S2 and S3). There were 2683 and 2432 overlapped DEGs in the three comparisons TD/TTR13/ETW vs. AT2 in leaves and young inflorescences respectively; however, the DEGs uniquely detected in ETW (leaves: 3415/42.43%; young inflorescences: 3877/46.63%) were nearly twice of the number in TD vs. AT2 (leaves: 1648/24.39%; young inflorescences: 1175/23.47%) and TTR13 vs. AT2 (leaves: 1302/20.34%; young inflorescences: 778/15.45%) (Figure S2). By dissecting DEGs into different genomes, we found genes in BB-genome were more likely to be differentially expressed than those in AA-genome in most comparisons (Table S4). We also classified DEGs into two regulatory directions, either upregulated or downregulated, and found no significant difference between the numbers of upregulated vs. downregulated DEGs in all comparisons in leaf tissue. However, in young inflorescences, the numbers of upregulated and downregulated DEGs were significantly different in all comparisons (binomial test *p*-value < 0.05) except two (TTR13 vs. AT2 and ETW vs. TTR13) (Table S5).

### 3.2. Enrichment of Gene Functions/Families in DEGs

**Figure 3:**
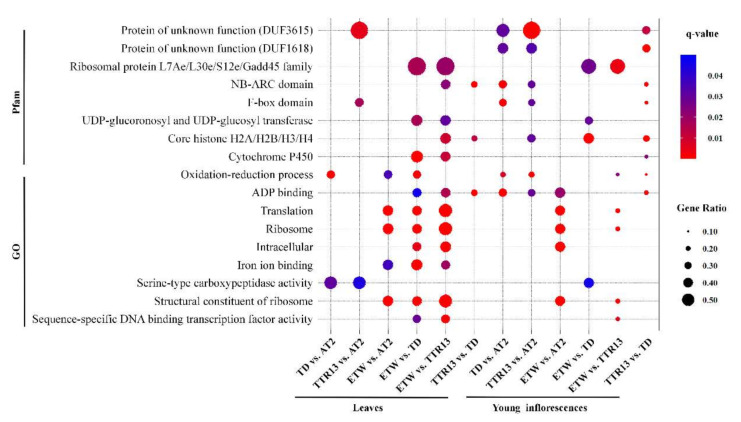
Functional enrichment of DEGs. GO (gene ontology) (lower) or Pfam (upper) terms that were significantly (*q*-value < 0.05) over-represented in DEGs of three or more comparisons are shown. The dot sizes correspond to proportions of DEGs and colors correspond to the *q*-values.

To explore the relationship between expression changes and gene function, we performed enrichment analysis of DEGs in gene ontology (GO) and Pfam terms using a hyper-geometric method. The raw *p*-values were adjusted by the FDR method. A few GO and Pfam terms were found to be significantly over-represented in DEGs (*q*-value < 0.05), indicating functional relevance of the changes in gene expression between genotypes (Figure 3, Supporting Dataset 2). Some GO terms such as “Oxidation-reduction process” and “ADP-binding” were found to be over-represented in half or more of the comparisons (3/6, number of significant comparisons/number of total comparisons) in leaves and in young inflorescences (4/6); and some Pfam terms such as “NB-ARC domain” and “Core histone H2A/H2B/H3/H4” were also significantly over-represented in several comparisons: (2/6) in leaves and (3/6) in young inflorescences, respectively (Figure 3, Supporting Dataset 2). The DEGs between extracted wheat ETW and two natural tetraploid wheat (TD and TTR13) were also enriched in the transcription factor related GO term “sequence-specific DNA binding transcription factor activity”. Together, these GO and Pfam results suggest that genes in some functional classes or families are more likely than others to undergo changes in expression during the evolution of polyploid wheat.

### 3.3. Distribution of DEGs in Different Duplication Modes

All flowering plants have experienced at least two rounds of ancient whole genome duplication (WGD), leading to retained syntenic blocks within plant genomes. With this in mind, we used MCScanX [27] to place all A and B chromosomal genes into five categories based on their modes of duplication: singletons (no duplication), dispersed (duplicated but not in synteny), proximal (duplicated but separated by fewer than 20 genes), tandem (adjacent), and WGD (retained in synteny). We identified 3084 and 2179 ancient WGD genes in A and B genomes, respectively, in the recently released assembly of wild tetraploid emmer wheat (*T. turgidum* ssp. *dicoccoides*) [16] (Table S6).

**Figure 4:**
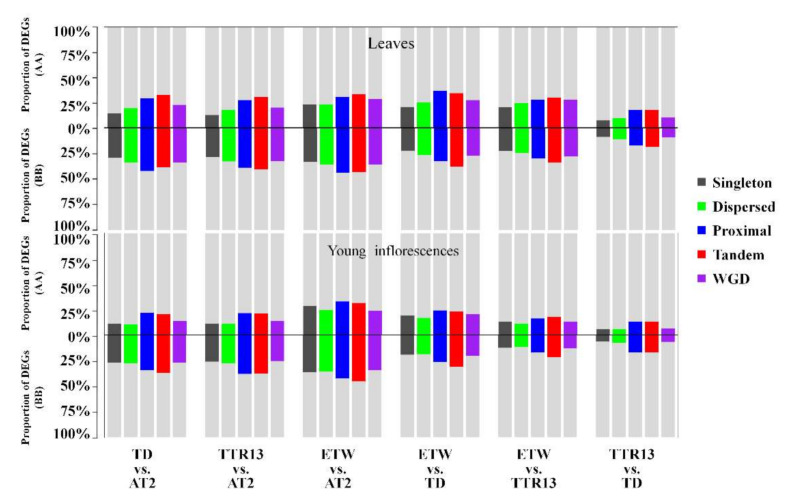
Proportion of DEGs in five duplication modes in different comparisons. The percentages of DEGs in AA and BB genome are shown on the up and down sides of *x*-axis. Different duplicate modes are shown in different colors in leaves (upper panel) and young inflorescences (lower panel) for each comparison.

To explore the relationship between models of duplication and changes in gene expression, we assessed the proportion of DEGs for each gene duplication mode. The proportion of DEGs in tandemly duplicated genes ranged from 13.31% to 34.73% in the A genome and from 17.02% to 45.39% in the B genome, and similar proportions, A: 13.56–37.01%; B: 16.97–44.12%, for proximally duplicated genes, across tissues. These two gene duplication modes showed a relatively higher proportion of DEGs than other modes in both genomes for each comparison (Figure 4, Table S7). Singleton genes had the smallest proportion of DEGs in all comparisons in leaves and two comparisons (TTR13 vs. AT2 and TTR13 vs. TD) in young inflorescences (Figure 4, Table S7). Dispersed and WGD duplicates showed slightly higher percentages of DEGs than singletons in all comparisons in leaves but lower percentages in four comparisons (TD vs. AT2, ETW vs. AT2, ETW vs. TD, and ETW vs. TTR13) in young.

### 3.4. Distribution of DEGs in A-B Homeolog Pairs

The A and B genomes share a common ancestor ~6.5 MYA, leading to a large number of orthologous genes between the two genomes, termed “homeologs” in tetraploids. To address changes in gene expression within and between the A and B genomes, we compared the DEGs and their homeologous genes as previously identified [16]. In total, 7682 A-B homeolog pairs were studied. Among them, 1496 (19.4%) to 5360 (69.8%) pairs in leaves and 968 (12.6%) to 5108 (66.5%) pairs in young inflorescences had at least one differentially expressed copy in comparisons between genotypes (Table 1). For these homeolog pairs with DEGs, most of them had only one differentially expressed copy between genotypes (DEG in A or B) and there were more DEGs in B homeologs than in A homeologs (Table 1). For homeolog pairs in which both copies were DEGs (DEG in both A and B), we classified them into two types, AB-convergent (both A and B downregulated or upregulated) and AB-divergent (A and B regulated in opposite directions). We found that homeolog pair with two DEGs were more likely to be AB-convergent than AB-divergent, especially in the comparisons between ETW and TD/TTR13, nearly all such pairs (>93%) were AB-convergent (Table 1).

### 3.5. Relationship between Gene Expression Changes and Selection

**Figure 5:**
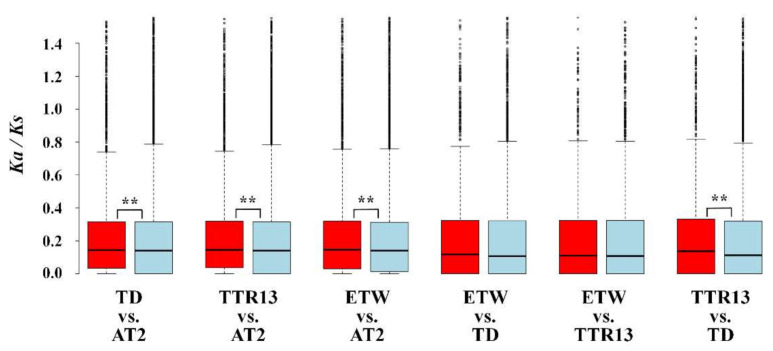
Distribution *Ka/Ks* ratios for genes in DEG category (red) and non-DEG category (blue). Any gene differentially expressed between genotypes in either tissue was grouped into DEG category, while, others were grouped into non-DEG category. The significance of Mann–Whitney–Wilcoxon test between DEG category and non-DEG category are shown as ** *p*-value < 0.01.

To explore the relationship between gene expression changes and selection pressures, we calculated *Ka/Ks* ratio for each gene. For clarity, genes were not differentially expressed between two genotypes in both tissues were grouped into non-DEG category, while genes differentially expressed in either tissue were grouped into the DEGs category. We compared the *Ka/Ks* ratios for DEG category and non-DEG category (Figure 5). Only a small proportion of genes had *Ka/Ks* ratios larger than 1, suggesting that these genes have likely experienced positive selection during wheat evolution. For most genes, the *Ka/Ks* ratios were less than 1 and the mean *Ka/Ks* ratio varied from 0.237 to 0.252 in DEG category and from 0.228 to 0.233 in non-DEG category indicating a predominant role of negative selection on these genes during the evolution of wheat species. However, overall *Ka/Ks* ratios in DEG category were elevated as compared to non-DEG category in all genotype pairs and were also statistically significant (Mann–Whitney–Wilcoxon Test, *p*-value < 0.01) except for ETW vs. TD and ETW vs. TTR13 (Table S8) that indicated a relaxed purifying selection on genes differentially expressed between genotypes.

## 4. Discussion

### 4.1. Effects of Polyploidization on the Evolution of Gene Expression

Changes in gene expression could happen by the onset of polyploidization due to genetic and epigenetic interactions and *de novo* changes which have been reported in newly synthesized or recently formed polyploid plants [37]. In polyploid wheat, rapid and substantial DNA loss has also been reported in very early generations of synthetics [38,39]. Recent genomics-based studies of bread wheat revealed that there was extensive gene loss in hexaploid wheat [40]. Moreover, other types of genetic changes, such as single nucleotide polymorphisms and gene copy number variation have been reported in different tetraploid wheat genotypes [41]. These genetic variations might have been responsible for the observed gene expression changes in different polyploid wheats. Along with genetic changes, heritable epigenetic changes have been reported followed allopolyploidization in wheat that may also have contributed to the transcriptome changes in the A and B subgenomes of polyploid wheat [42,43]. Indeed, in hexaploid bread wheat, it has been shown that permanent silencing of particular gene homeologs can be caused by altered DNA methylation [44]. The A and B genomes of ETW experienced one more polyploidization event and resided in the same hexaploid nucleus with the D genome for 8000–10,000 years before extraction. We found there were more DEGs in ETW than TD and TTR13 as compared to AT2 (4.76–6.08% and 12.02–11.90% more DEGs in leaves and young inflorescences) (Figure 2 and Table S2). Our results indicate that the period of hexaploidy may have resulted in accelerated changes in gene expression and led to many irreversible expression alterations in both the A and B genomes as compared to the natural and domesticated BBAA tetraploid TD and TTR13, which is consistent with an earlier study based on microarray analysis [10]. Although the extraction process of ETW from hexaploid involves a serious of backcrosses and repeated changes of ploidy could introduce some new genetic and/or epigenetic mutations, there is no evidence that these processes can lead to enormous gene expression changes [10]. Moreover, these DEGs were found to be enriched in some GO or Pfam terms indicating that they had functional preferences rather than resulted from the random changes due to the extraction process. Furthermore, we found that *Ka/Ks* ratios of genes in DEG category were higher than those in non-DEG category in TD vs. AT2, TTR13 vs. AT2 and ETW vs. AT2, indicating that these DEGs experienced relaxed purifying selection. As a matter of fact, the AA and BB genomes in AT2 had evolved in diploid form before they are artificially synthesized together, however, the two genomes in the rest BBAA genotypes have evolved in polyploid status since the onset of the common ancestor of tetraploid wheat (Figure 1). The polyploidization process leads to gene duplication and functional redundancy. The neo-/non-functionalization hypothesis predicts that one duplicated gene copy can maintain the ancestral function while the other copy is relaxed from purifying selection [45]. Here, our results suggest that some differentially expressed genes are likely experienced relaxed purifying selection in the evolution of wheat following allopolyploidization.

### 4.2. Mode of Ancient Gene Duplication Is Related to Gene Expression Changes

Duplicated genes from the five modes (singleton, dispersed, proximal, tandem, and WGD/segmental duplicates) identified in the A or B genome are mostly the legacies of ancient WGDs and/or subsequent gene duplication/diploidization events that occurred before divergence of A and B genomes [1,2]. Recent studies have shown that genes in different functional categories tend to have different evolutionary fates after WGDs [23,25,46]. For example, some housekeeping genes and nuclear-encoded organellar (chloroplast and mitochondria) genes commonly revert to singleton status after duplication [23,46]. In contrast, transcription factor genes are more likely to be retained as duplicate after WGDs [25]. These patterns can be explained by the gene balance hypothesis and its amendment, which posits that stoichiometric or dosage balances of gene products that interact in networks and functional pathways with other genes are more constrained to lose copies [47,48]. Tandem duplication, on the other hand, is a duplication mechanism distinct from WGD, and thus tandemly duplicated genes have been proposed to be dosage-insensitive and less regulated by gene balance [49]. Therefore, genes in different duplicate modes may be subject to different evolutionary constraints following duplication. Here, we observed that the proportions of DEGs varied across duplication modes. Singleton and dispersed duplicate genes contained lower proportions of DEGs than other types, indicating genes in these duplicate modes were more stable in expression during evolution of different wheat species. Meanwhile, a higher proportion of DEGs was found for tandemly duplicated genes and proximal genes in all comparisons, suggesting that these genes experienced faster expression changes, through copy number variation and/or the accumulation of genetic and epigenetic regulatory mutations. These results indicate that the duplication modes might also affect the changes of the expression of duplicated genes during polyploid wheat evolution.

### 4.3. Cis- and Inter-Subgenome Trans-Regulatory Changes Play Roles in the Evolution of Gene Expression in Wheat

Gene and genome duplication have been shown to play important roles in the evolution of regulatory networks [50,51]. Duplicated genes can undergo rapid expression divergence following WGDs thus further increase the complexity of their regulatory networks [51]. In tetraploid wheat, both genes in a homeolog pair would be regulated in the same direction (up or down; convergent) if they are due to changes in expression or function of an inter-subgenome *trans*-acting factor; in contrast, a *cis*-regulatory change should affect the expression of one copy only. We observed that for A-B homeolog pairs of DEGs, most of them (>80%) had only one differentially expressed copy from A or B genome (DEG in A or B), indicating that either expression changes of these genes was due to mutations in *cis*-acting regions or these A and B homeologs were regulated by distinct networks (Table 1). Of the homeolog pairs in which both homeologs (DEG in A and B) were DEGs, we observed more AB-convergent genes (both up or both down) than AB-divergent genes, particularly in comparisons between natural tetraploid (TD, TTR13) and the extracted tetraploid ETW where almost all (>93%) were modulated in the same direction. This suggests that most differentially expressed homeolog pairs (DEG in A and B) are likely due to changes in *trans*-regulatory factors affecting both A and B-subgenome, which accords with the observation that a transcription factor related GO term were significantly over-represented in DEGs of ETW vs. TD and ETW vs. TTR13. Together, our results indicate that changes in both *cis*- and inter-subgenome *trans*-regulation playing roles in the expression evolution of homeologous gene copies during the evolution of wheat at different ploidy levels.

## 5. Conclusions

In this study, homeologous gene expression changes were investigated in four BBAA tetraploid wheats in which the AA and BB genomes, either separately or together, came from three different ploidy backgrounds. Our results indicate that both gene functions and duplication modes are important factors contributing to the evolution of homeologous gene expression in polyploid wheat, with polyploidization events playing a major role primarily via relaxed purifying selection on redundant genes and inter-subgenome *trans*- regulation. Our results have shed new light on the post-polyploidization evolution in wheat.

## Figures and Tables

**Table 1:** Distribution and regulatory types of DEGs in A-B homeolog pairs.

	TD vs. AT2	TTR13 vs. AT2	ETW vs. AT2	ETW vs. TD	ETW vs. TTR13	TTR13 vs. TD
Leaves ^a^	4418	4136	5360	4694	4651	1496
DEG in A or B ^b^	3839	3646	4502	3788	3715	1363
A-DEG	1312	1206	1806	1841	1817	620
B-DEG	2527	2440	2696	1947	1898	743
DEG in A and B ^c^	579	490	858	906	936	133
AB-convergent	416	330	671	846	892	105
AB-divergent	163	160	187	60	44	28
Young inflorescences ^a^	2866	2944	5108	3071	1873	968
DEG in A or B ^b^	2621	2675	4352	2543	1598	879
A-DEG	629	645	1648	1169	730	372
B-DEG	1992	2030	2704	1374	868	507
DEG in A and B ^c^	245	269	756	528	275	89
AB-convergent	138	151	588	494	259	69
AB-divergent	107	118	168	34	16	20

^a^—the number of A-B homeolog pairs with one or two DEGs between genotypes. ^b^—the number of A-B homeolog pairs with only one DEG between genotypes. ^c^—the number of A-B homeolog pairs in which both copies were DEGs between genotypes.

## Supplementary Materials

The following are available online at https://www.mdpi.com/2073-4425/11/12/1401/s1. Figure S1. Dendrogram based on the cluster of gene expression for different replicates in different tissues and genotypes. Figure S2. Venn-diagram of DEGs among TD/TTR13/ETW vs. AT2. Table S1. Mapping statistics of RNA-seq data. Table S2. Numbers and regulatory types of DEGs in each comparison. Table S3. Pairwise chi-square test of DEG proportions between different comparisons. Table S4. Number and percentage of AA and BB genes differentially expressed in each comparison. Table S5. Number of upregulated and downregulated DEGs in each comparison. Binomial test p-value between upregulated and downregulated DEGs are shown for each comparison. Table S6, Number of genes in the five duplicate modes in AA and BB genome. Table S7, Numbers and proportions of DEGs in five gene categories (singleton, dispersed, proximal, tandem, and WGD) in each comparison in leaves and young inflorescences. Table S8, Summary of Ka/Ks ratios for DEGs and Non-DEGs in each comparison. Supporting Dataset 1: List of DEGs in each comparison. Supporting Dataset 2: List of all over-represented GO/Pfam terms in each comparison.
