# Supplementary material for: Evolution of Homeologous Gene Expression in Polyploid Wheat
Source: Genes (Basel). 2020 Nov 25;11(12):1401. doi: 10.3390/genes11121401 (PMC7759873; doi:10.3390/genes11121401)

**Figure S1. Dendrogram based on the cluster of gene expression for different replicates in different tissues and genotypes.** Two biological replicates of each wheat genotype were used, genotypes from different ploidy backgrounds were marked by different colors: diploid, yellow; tetraploid, red; hexaploid, orange.


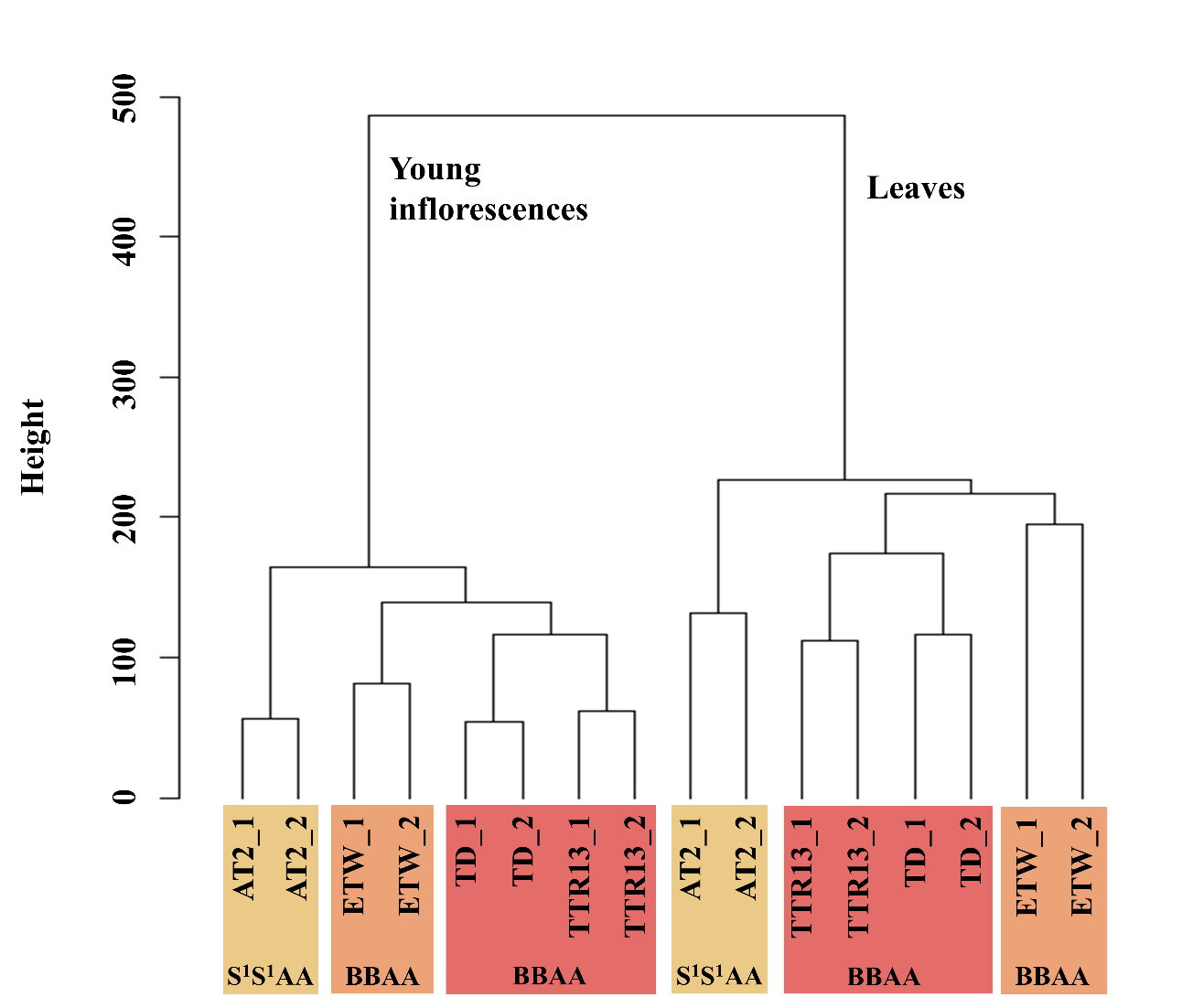

Supplement: Supplementary file 1 [file genes-11-01401-s001.zip › Figure S1.docx]
