# Supplementary material for: Evolution of Homeologous Gene Expression in Polyploid Wheat
Source: Genes (Basel). 2020 Nov 25;11(12):1401. doi: 10.3390/genes11121401 (PMC7759873; doi:10.3390/genes11121401)
Supplement: Supplementary file 1 [file genes-11-01401-s001.zip › Table S1.docx]

Table S1. Mapping statistics of RNA-seq data.

| **Sample** | **Number of input reads** | **Average input read length** | **Uniquely mapped reads** |
| --- | --- | --- | --- |
| AT2_L_1 | 26,609,683 | 15,920,376 | 59.83% |
| AT2_L_2 | 24,699,306 | 17,899,038 | 72.47% |
| AT2_S_1 | 36,432,271 | 22,760,694 | 62.47% |
| AT2_S_2 | 37,454,549 | 27,910,190 | 74.52% |
| Tdi_L_1 | 37,712,569 | 25,006,088 | 66.31% |
| Tdi_L_2 | 41,210,870 | 22,768,938 | 55.25% |
| Tdi_S_1 | 34,648,885 | 27,758,440 | 80.11% |
| Tdi_S_2 | 38,381,058 | 29,562,660 | 77.02% |
| TTR13_L_1 | 32,727,274 | 26,168,253 | 79.96% |
| TTR13_L_2 | 29,309,795 | 22,907,076 | 78.16% |
| TTR13_S_1 | 41,559,615 | 29,505,872 | 71.00% |
| TTR13_S_2 | 39,062,205 | 29,914,216 | 76.58% |
| ETW_L_1 | 30,000,000 | 25,806,183 | 86.02% |
| ETW_L_2 | 30,000,000 | 24,574,122 | 81.91% |
| ETW_S_1 | 30,000,000 | 26,293,177 | 87.64% |
| ETW_S_2 | 30,000,000 | 25,782,294 | 85.94% |

The RNA-seq data were from a previously published study by Wang et al. 2016 (PRJNA272886). All raw RNA-seq data were mapped to the genome sequences of a tetraploid wheat (Triticum turgidum ssp. dicoccoides, “emmer wheat”) using STAR
