# Supplementary material for: Evolution of Homeologous Gene Expression in Polyploid Wheat
Source: Genes (Basel). 2020 Nov 25;11(12):1401. doi: 10.3390/genes11121401 (PMC7759873; doi:10.3390/genes11121401)
Supplement: Supplementary file 1 [file genes-11-01401-s001.zip › Table S2.docx]

**Table S2. Numbers and regulatory types of DEGs in each comparison.**

| **Leaves** | | | | | | |
| --- | --- | --- | --- | --- | --- | --- |
|  | **TD vs. AT2** | **TTR13 vs. AT2** | **ETW vs. AT2** | **ETW vs. TD** | **ETW vs. TTR13** | **TTR13 vs. TD** |
| AA up-regulated | 1,618 | 1,458 | 2,160 | 2,079 | 2,027 | 813 |
| BB up-regulated | 1,802 | 1,748 | 1,918 | 1,434 | 1,380 | 579 |
| AA down-regulated | 1,624 | 1,502 | 2,107 | 2,127 | 2,018 | 855 |
| BB down-regulated | 1,713 | 1,692 | 1,864 | 1,371 | 1,268 | 577 |
| **Total** | 6,757 (24.94%) | 6,400 (23.62%) | 8,049 (29.70%) | 7,011 (25.87%) | 6,693 (24.70%) | 2,824 (10.42%) |
| **Young inflorescences** | | | | | | |
|  | **TD vs. AT2** | **TTR13 vs. AT2** | **ETW vs. AT2** | **ETW vs. TD** | **ETW vs. TTR13** | **TTR13 vs. TD** |
| AA up-regulated | 1,061 | 1,141 | 2,203 | 1,584 | 1,084 | 611 |
| BB up-regulated | 1,297 | 1,333 | 1,744 | 1,136 | 682 | 510 |
| AA down-regulated | 987 | 944 | 2,201 | 1,500 | 985 | 504 |
| BB down-regulated | 1,661 | 1,619 | 2,115 | 966 | 666 | 331 |
| **Total** | 5,006 (18.47%) | 5,037 (18.59%) | 8,263 (30.49%) | 5,186 (19.14%) | 3,417 (12.61%) | 1,956 (7.22%) |
