# Supplementary material for: Evolution of Homeologous Gene Expression in Polyploid Wheat
Source: Genes (Basel). 2020 Nov 25;11(12):1401. doi: 10.3390/genes11121401 (PMC7759873; doi:10.3390/genes11121401)
Supplement: Supplementary file 1 [file genes-11-01401-s001.zip › Table S3.docx]

**Table S3. Pairwise chi-square test of DEG proportions between different comparisons.** P-values for comparisons in leaves (green) and young inflorescences (red) are shown in different colors.

| **L**  **YI** | **TD vs. AT2** | **TTR13 vs. AT2** | **ETW vs. AT2** | **ETW vs. TD** | **ETW vs. TTR13** | **TTR13 vs. TD** |
| --- | --- | --- | --- | --- | --- | --- |
| **TD vs. AT2** | 1 | 0.0003616 | 2.20E-16 | 0.01254 | 0.531 | 2.20E-16 |
| **TTR13 vs. AT2** | 0.7401 | 1 | 2.20E-16 | 1.26E-09 | 0.003386 | 2.20E-16 |
| **ETW vs. AT2** | 2.20E-16 | 2.20E-16 | 1 | 2.20E-16 | 2.20E-16 | 2.20E-16 |
| **ETW vs. TD** | 0.0491 | 0.1042 | 2.20E-16 | 1 | 0.001731 | 2.20E-16 |
| **ETW vs. TTR13** | 2.20E-16 | 2.20E-16 | 2.20E-16 | 2.20E-16 | 1 | 2.20E-16 |
| **TTR13 vs. TD** | 2.20E-16 | 2.20E-16 | 2.20E-16 | 2.20E-16 | 2.20E-16 | 1 |
