# Supplementary material for: Evolution of Homeologous Gene Expression in Polyploid Wheat
Source: Genes (Basel). 2020 Nov 25;11(12):1401. doi: 10.3390/genes11121401 (PMC7759873; doi:10.3390/genes11121401)
Supplement: Supplementary file 1 [file genes-11-01401-s001.zip › Table S4.docx]

**Table S4. Number and percentage of AA and BB genes differentially expressed in each comparison.**

16,526 and 10,571 genes from A and B genomes were analyzed, respectively.

| **Leaves** | | | | | | |
| --- | --- | --- | --- | --- | --- | --- |
|  | **TD vs. AT2** | **TTR13 vs. AT2** | **ETW vs. AT2** | **ETW vs. TD** | **ETW vs. TTR13** | **TTR13 vs. TD** |
| DEGs in AA | 3,242/19.6% | 2,960/17.9% | 4,267/25.8% | 4,206/25.5% | 4,045/24.5% | 1,668/10.1% |
| DEGs in BB | 3,515/33.3% | 3,440/32.5% | 3,782/35.8% | 2,805/26.5% | 2,648/25.0% | 1,156/10.9% |
| **p-value (chi-squared test)** | 2.2e-16 | 2.2e-16 | 2.2e-16 | 0.048 | 0.292 | 0.028 |
| **Young inflorescences** | | | | | | |
|  | **TD vs. AT2** | **TTR13 vs. AT2** | **ETW vs. AT2** | **ETW vs. TD** | **ETW vs. TTR13** | **TTR13 vs. TD** |
| DEGs in AA | 2,048/12.4% | 2,085/12.6% | 4,404/26.6% | 3,084/18.7% | 2,069/12.5% | 1,115/6.7% |
| DEGs in BB | 2,958/28.0% | 2,952/27.9% | 3,859/36.5% | 2,102/19.9% | 1,348/12.8% | 841/8.0% |
| **p-value ( chi-squared test)** | 2.2e-16 | 2.2e-16 | 2.2e-16 | 0.013 | 0.587 | 1.94e-4 |
