# Supplementary material for: Evolution of Homeologous Gene Expression in Polyploid Wheat
Source: Genes (Basel). 2020 Nov 25;11(12):1401. doi: 10.3390/genes11121401 (PMC7759873; doi:10.3390/genes11121401)
Supplement: Supplementary file 1 [file genes-11-01401-s001.zip › Table S5.docx]

**Table S5.** **Number of up- and down-regulated DEGs in each comparison**. Binomial test p-value between up and down-regulated DEGs are shown for each comparison.

| **Leaves** | | | | | | |
| --- | --- | --- | --- | --- | --- | --- |
|  | **TD vs. AT2** | **TTR13 vs. AT2** | **ETW vs. AT2** | **ETW vs. TD** | **ETW vs. TTR13** | **TTR13 vs. TD** |
| DEGs up_regulated | 3,420 | 3,206 | 4,078 | 3,513 | 3,407 | 1,392 |
| DEGs down_regulated | 3,337 | 3,194 | 3,971 | 3,498 | 3,286 | 1,432 |
| **p-value (binomial test)** | 0.3185 | 0.8906 | 0.2374 | 0.8672 | 0.1424 | 0.463 |
| **Young inflorescences** | | | | | | |
|  | **TD vs. AT2** | **TTR13 vs. AT2** | **ETW vs. AT2** | **ETW vs. TD** | **ETW vs. TTR13** | **TTR13 vs. TD** |
| DEGs up_regulated | 2,358 | 2,474 | 3,947 | 2,720 | 1,766 | 1,121 |
| DEGs down_regulated | 2,648 | 2,563 | 4,316 | 2,466 | 1,651 | 835 |
| **p-value (binomial test)** | 4.395e-05 | 0.215 | 5.144e-05 | 0.0004417 | 0.05113 | 1.08e-10 |
