# Supplementary material for: Evolution of Homeologous Gene Expression in Polyploid Wheat
Source: Genes (Basel). 2020 Nov 25;11(12):1401. doi: 10.3390/genes11121401 (PMC7759873; doi:10.3390/genes11121401)
Supplement: Supplementary file 1 [file genes-11-01401-s001.zip › Table S6.docx]

**Table S6. Number of genes in the five duplicate modes in AA and BB genome.**

|  | **AA genome** | | **BB genome** | |
| --- | --- | --- | --- | --- |
|  | **No. of Genes** | **Expressed Genes** | **No. of Genes** | **Expressed Genes** |
| **Singleton** | 19,337 | 4,556 (23.56%) | 21,068 | 2,373 (11.26%) |
| **Dispersed** | 22,751 | 9,011 (39.61%) | 24,392 | 6,469 (26.52%) |
| **Proximal** | 3,287 | 708 (21.54%) | 4,048 | 442 (10.92%) |
| **Tandem** | 3,175 | 714 (22.49%) | 3,670 | 423 (11.53%) |
| **WGD** | 3,084 | 1,537 (49.84%) | 2,179 | 864 (39.65%) |
