# Supplementary material for: Evolution of Homeologous Gene Expression in Polyploid Wheat
Source: Genes (Basel). 2020 Nov 25;11(12):1401. doi: 10.3390/genes11121401 (PMC7759873; doi:10.3390/genes11121401)
Supplement: Supplementary file 1 [file genes-11-01401-s001.zip › Table S7.docx]

**Table S7. Numbers and proportions of DEGs in five gene categories (singleton, dispersed, proximal, tandem, and WGD) in each comparison in leaves and young inflorescences.**

| **Leaves** | | | | | | | | | | |
| --- | --- | --- | --- | --- | --- | --- | --- | --- | --- | --- |
|  | **Singleton** | | **Dispersed** | | **Proximal** | | **Tandem** | | **WGD** | |
|  | **A** (4,556) | **B** (2,373) | **A** (9,011) | **B** (6,469) | **A** (708) | **B** (442) | **A** (714) | **B** (423) | **A** (1,537) | **B** (864) |
| **TD** vs. **AT2** | 656  (14.40%) | 698  (29.41%) | 1,786  (19.82%) | 2,176  (33.64%) | 209  (29.52%) | 187  (42.31%) | 235  (32.91%) | 162  (38.30%) | 356  (23.16%) | 292  (33.80%) |
| **TTR13** vs. **AT2** | 591 (12.97%) | 684  (28.82%) | 1,638  (18.18%) | 2,131  (32.94%) | 197  (27.82%) | 172  (38.91%) | 221  (30.95%) | 172  (40.66%) | 313  (20.36%) | 281  (32.52%) |
| **ETW** vs. **AT2** | 1,063  (23.33%) | 785  (33.08%) | 2,296  (25.48%) | 2,309  (35.69%) | 219  (30.93%) | 195  (44.12%) | 240  (33.61%) | 182  (43.03%) | 449  (29.21%) | 311  (36.00%) |
| **ETW** vs. **TD** | 962  (21.12%) | 530  (22.33%) | 2,306  (25.59%) | 1,735  (26.82%) | 262  (37.01%) | 143  (32.35%) | 248  (34.73%) | 161  (38.06%) | 428  (27.85%) | 236  (27.31%) |
| **ETW** vs. **TTR13** | 944  (20.72%) | 534  (22.50%) | 2,253  (25.00%) | 1,597  (24.69%) | 199  (28.11%) | 132  (29.86%) | 217  (30.39%) | 144  (34.04%) | 432  (28.11%) | 241  (27.89%) |
| **TTR13** vs. **TD** | 344  (7.55%) | 208  (8.77%) | 899  (9.98%) | 711  (10.99%) | 129  (18.22%) | 77  (17.42%) | 130  (18.21%) | 79  (18.68%) | 166  (10.80%) | 81  (9.38%) |
| **Young inflorescences** | | | | | | | | | | |
|  | **Singleton** | | **Dispersed** | | **Proximal** | | **Tandem** | | **WGD** | |
|  | **A** (4,556) | **B** (2,373) | **A** (9,011) | **B** (6,469) | **A** (708) | **B** (442) | **A** (714) | **B** (423) | **A** (1,537) | **B** (864) |
| **TD** vs. **AT2** | 529  (11.61%) | 644  (27.14%) | 993  (11.02%) | 1,774  (27.42%) | 157  (22.18%) | 151  (34.16%) | 150  (21.01%) | 156  (36.88%) | 219  (14.25%) | 233  (26.97%) |
| **TTR13** vs. **AT2** | 524  (11.50%) | 620  (26.13%) | 1,035  (11.49%) | 1,784  (27.58%) | 154  (21.75%) | 168  (38.01%) | 153  (21.43%) | 160  (37.83%) | 219  (14.25%) | 220  (25.46%) |
| **ETW** vs. **AT2** | 1,318  (28.93%) | 863  (36.37%) | 2,253  (25.00%) | 2,319  (35.85%) | 234  (33.05%) | 187  (42.31%) | 226  (31.65%) | 192  (45.39%) | 373  (24.27%) | 298  (34.49%) |
| **ETW** vs. **TD** | 880  (19.32%) | 459  (19.34%) | 1,542  (17.11%) | 1,218  (18.83%) | 173  (24.44%) | 117  (26.47%) | 166  (23.25%) | 132  (31.21%) | 323  (21.01%) | 176  (20.37%) |
| **ETW** vs. **TTR13** | 621  (13.63%) | 302  (12.73%) | 999  (11.09%) | 767  (11.86%) | 117  (16.53%) | 76  (17.19%) | 128  (17.93%) | 92  (21.75%) | 204  (13.27%) | 111  (12.85%) |
| **TTR13** vs. **TD** | 263  (5.77%) | 146  (6.15%) | 554  (6.15%) | 489  (7.56%) | 96  (13.56%) | 75  (16.97%) | 95  (13.31%) | 72  (17.02%) | 107  (6.96%) | 59  (6.83%) |
