# Supplementary material for: Evolution of Homeologous Gene Expression in Polyploid Wheat
Source: Genes (Basel). 2020 Nov 25;11(12):1401. doi: 10.3390/genes11121401 (PMC7759873; doi:10.3390/genes11121401)
Supplement: Supplementary file 1 [file genes-11-01401-s001.zip › Table S8.docx]

**Table S8. Summary of *Ka*/*Ks* ratios for DEGs and Non-DEGs in each comparison**. P-values between DEG category and non-DEG category were calculated using Mann-Whitney-Wilcoxon test.

|  | **DEG** | | **Non-DEG** | |  |
| --- | --- | --- | --- | --- | --- |
|  | **Gene number** | **mean *Ka*/*Ks*** | **Gene number** | **mean *Ka*/*Ks*** | **p-value** |
| **TD** vs. **AT2** | 4,639 | 0.247 | 12,219 | 0.233 | 9.64E-03 |
| **TTR13** vs. **AT2** | 4,446 | 0.252 | 12,424 | 0.230 | 1.30E-03 |
| **ETW** vs. **AT2** | 5,307 | 0.246 | 11,572 | 0.233 | 7.65E-03 |
| **ETW** vs. **TD** | 2,507 | 0.237 | 6,578 | 0.228 | 0.2089049 |
| **ETW** vs. **TTR13** | 1,327 | 0.231 | 3,574 | 0.226 | 0.3449536 |
| **TTR13** vs. **TD** | 1,127 | 0.259 | 8,090 | 0.224 | 6.57E-04 |
