# Supplementary figures and images for: Evolution of Homeologous Gene Expression in Polyploid Wheat
Source: Genes (Basel). 2020 Nov 25;11(12):1401. doi: 10.3390/genes11121401 (PMC7759873; doi:10.3390/genes11121401)

**Figure S2. Venn-diagram of DEGs among TD/TTR13/ETW vs. AT2.**


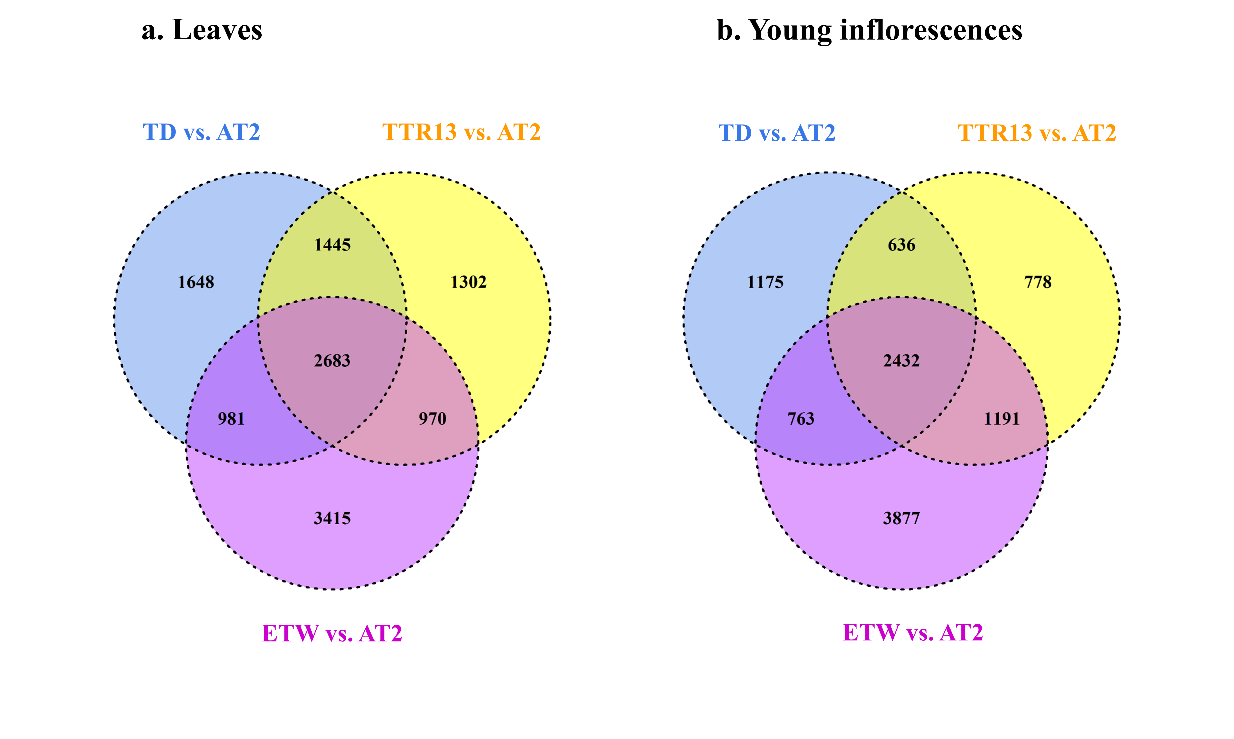

Supplement: Supplementary file 1 [file genes-11-01401-s001.zip › Figure S2.docx]
